# Supplementary material for: Dietary Potassium and Clinical Outcomes among Patients on Peritoneal Dialysis
Source: Nutrients. 2023 Oct 6;15(19):4271. doi: 10.3390/nu15194271 (PMC10574508; doi:10.3390/nu15194271)
Supplement: Supplementary file 1 [file nutrients-15-04271-s001.zip › nutrients-2602289-supplementary.pdf]

Table S1. Clinical characteristics of peritoneal dialysis patients according to time-averaged dietary potassium (*n* = 881)

**Characteristic**	**Time-averaged dietary potassium**		***p***
	**<1200 mg/d**	**≥1200 mg/d**	
Age, years Male,	60.3 ± 14.9 [1]	56.5 ± 14.6	< 0.001
n (%) BMI [2], kg/m^2	103 (35.6)	331 (55.9)	< 0.001
Educational level	22.8 ± 4.0	23.4 ± 3.5	0.031
			< 0.001
≤Elementary school, n (%)	76 (26.3)	73 (12.3)	
Middle school, n (%)	82 (28.4)	147 (24.8)	
High school, n (%)	71 (24.6)	173 (29.2)	
>High school, n (%)	60 (20.8)	199 (33.6)	
DM, n (%)	142 (49.1)	229 (38.7)	0.003
CV disease, n (%)	140 (48.4)	235 (39.7)	0.014
SBP, mmHg	133.9 ± 17.7	134.2 ± 14.1	0.854
DBP, mmHg	76.7 ± 10.5	79.0 ± 10.1	0.003
MAP, mmHg	96.8 ± 11.6	98.2 ± 10.6	0.075
Laboratory and nutritional data			
Albumin, g/L	34.7 ± 4.3	36.5 ± 3.9	< 0.001
Hemoglobin, g/L	107.9 ± 15.1	109.9 ± 13.8	0.068
Hs-CRP, mg/L	5.7 (2.2, 12.8)	3.4 (1.3, 7.3)	< 0.001
Urea nitrogen, mmol/L	20.2 ± 5.1	22.6 ± 4.6	< 0.001
Serum creatinine, μmol/L	737.0 ± 222.4	812.2 ± 254.0	< 0.001
Serum calcium, mmol/L	2.3 ± 0.4	2.3 ± 0.3	0.361
Serum phosphorus, mmol/L	1.5 ± 0.4	1.6 ± 0.3	0.001
Serum potassium, mmol/L	4.2 ± 0.5	4.5 ± 0.5	< 0.001
Serum sodium, mmol/L	138.5 ±3.6	138.9 ±2.5	0.060
HDL-cholesterol, mmol/L	1.1 ± 0.3	1.1 ± 0.3	0.431
LDL-cholesterol, mmol/L	2.7 ± 0.7	2.6 ± 0.7	0.021
Total cholesterol, mmol/L	5.0 ± 1.1	4.8 ± 1.9	0.037
Triglycerides, mmol/L	1.7 (1.3, 2.4)	1.6 (1.3, 2.3)	0.480
iPTH, pg/mL	136.4 (76.7, 233.8)	147.8 (79.1, 249.0)	0.505
Total CCr, L/w/1.73 m^2	60.3 ± 16.6	63.5 ± 18.5	0.014
Total Kt/V	1.9 ± 0.4	1.9 ± 0.4	0.917
RRF, ml/min	2.5 (1.5, 3.8)	2.8 (1.7, 4.6)	0.010
Energy intake, kcal/day	1117.7 ± 193.5	1473.7 ± 248.1	< 0.001
Protein intake, g/day	39.7 ± 8.9	54.2 ± 9.9	< 0.001
Fat intake, g/day	45.1 ± 10.5	57.7 ± 11.9	< 0.001
Carbohydrate intake, g/day	141.3 ± 30.0	191.2 ± 41.4	< 0.001
Fiber intake, g/day	5.5 ± 1.7	8.7 ± 2.8	< 0.001
Fatty acid intake, g/day	43.1 ± 10.6	43.9 ± 11.1	0.475
Potassium intake, mg/day	989.4 ± 161.4	1558.7 ± 297.0	< 0.001
nDEI, kcal/kg/d	24.0 ± 5.5	27.1 ± 5.7	< 0.001
nDPI, g/kg/d	0.7 ± 0.2	0.9 ± 0.2	< 0.001

[1] Values are expressed as mean ± standard deviation, percentage or median with upper and lower quartile or percentage. [2] Abbreviation: BMI, body mass index; CV, cardiovascular; CCr, creatinine clearance; DM, diabetes mellitus; DBP, diastolic blood pressure; Hs-CRP, high-sensitivity C-reactive protein; HDL, high-density lipoprotein; iPTH, intact parathyroid hormone; Kt/V, urea clearance; LDL, low-density lipoprotein; MAP, mean arterial pressure; nDEI, normalized energy intake; nDPI, normalized protein intake; RRF, residual renal function; SBP, systolic blood pressure.
